# Sensory Attenuation Assessed by Sensory Evoked Potentials in Functional Movement Disorders

**DOI:** 10.1371/journal.pone.0129507

**Published:** 2015-06-19

**Authors:** Antonella Macerollo, Jui-Cheng Chen, Isabel Pareés, Panagiotis Kassavetis, James Morvan Kilner, Mark John Edwards

**Affiliations:** 1 Sobell Department of Motor Neuroscience and Movement Disorders, The National Hospital of Neurology and Neurosurgery, Institute of Neurology, University College London, London, United Kingdom; 2 Department of Neuroscience and Sense Organs, Aldo Moro University of Bari, Bari, Italy; Centre Hospitalier Universitaire Vaudois Lausanne - CHUV, UNIL, SWITZERLAND

## Abstract

**Background:**

Functional (psychogenic) movement disorders (FMD) have features associated with voluntary movement (e.g. distractibility) but patients report movements to be out of their control. One explanation for this phenomenon is that sense of agency for movement is impaired. The phenomenon of reduction in the intensity of sensory experience when movement is self-generated and a reduction in sensory evoked potentials (SEPs) amplitude at the onset of self-paced movement (sensory attenuation) have been linked to sense of agency for movement.

**Methods:**

We compared amplitude of SEPs from median nerve stimulation at rest and at the onset of a self-paced movement of the thumb in 17 patients with FMD and 17 healthy controls.

**Results:**

Patients showed lack of attenuation of SEPs at the onset of movement compared to reduction in amplitude of SEPs in controls. FMD patients had significantly different ratios of movement onset to rest SEPs than did healthy controls at each electrode: 0.79 in healthy controls and 1.35 in patients at F3 (t = -4.22, p<0.001), 0.78 in healthy controls and 1.12 at patients C3 (t = -3.15, p = 0.004) and 0.77 in healthy controls and 1.05 at patients P3 (t = -2.88, p = 0.007).

**Conclusions:**

Patients with FMD have reduced sensory attenuation as measured by SEPs at onset of self-paced movement. This finding can be plausibly linked to impairment of sense of agency for movement in these patients.

## Introduction

Functional movement disorders (FMD) are part of the spectrum of functional neurological symptoms, one of the most frequent diagnoses made in neurological practice [1]. FMD are characterised by movements that require attention to manifest and conversely disappear with distraction. These characteristics would be expected in movements that were voluntarily produced, but patients report movements to be out of their control. There is still a pervasive view amongst neurologists that such patients are often feigning their symptoms, and this in part may explain the lack of interest in managing such patients, despite the common and disabling nature of functional symptoms.

A key issue therefore in understanding the nature and pathophysiology of FMD is the study of sense of agency. This would be expected to be impaired given patients’ report of lack of control over what appear to be voluntary movements. Sensory attenuation (SA) describes a phenomenon associated with normal movement where there is a different perception of identical sensory inputs depending on whether they are self-generated or externally generated [2].Stimuli which are self-generated are associated with a reduction in the perceived intensity of the stimulus; for example while one cannot tickle oneself, one can be tickled by others [3–8]. SA is proposed to be a characteristic of voluntary movement, and importantly it has been proposed as an implicit measure of the sense of agency for movement. Recently, we have demonstrated abnormal SA in patients with FMD. We used a classic “force matching” paradigm where subjects are asked to match a force delivered to their finger either by pressing down directly on their finger or operating a robot to press down on their finger [9]. This requires a complex experimental set-up and we were interested in developing a protocol that could probe the same aspect of movement control but in a simpler way and thus could be more suitable as a potential biomarker for FMD for future clinical studies. Here, we report the results of a study examining suppression of sensory evoked potentials (SEPs) at the onset of self-generated movements in healthy participants and FMD patients. We hypothesised that patients with FMD would have less SEPs suppression at the onset of movement compared to healthy controls.

## Material and Methods

### Participants

Seventeen patients with FMD affecting body parts excluding upper limbs were recruited from outpatient clinics at The National Hospital for Neurology and Neurosurgery, Queen Square, London, UK. They had documented or clinically established FMD following Fahn and Williams criteria [10]. Patients with sensory abnormalities were excluded. Seventeen healthy volunteers matched with respect of gender, age and handedness were studied as the control group (Table 1). The study was approved by the local institutional ethics committee. Written consent was obtained from all participants according to the declaration of Helsinki.

**Table 1:** Demographic and Clinical profile of the Participants.

	Patients	Healthy Control	p-value
**Age (years)**			
Mean (± Standard Deviation)	45.5 (±7.6)	48 (±7.4)	0.14
**Sex (n)**			
Male	5	7	0.45
Female	12	10	0.45
**Handness**			
Right	17	17	0.33
**Main functional symptoms (n)**			
Fixed dystonia of limbs	5	NA	
Functional gait impairment	7	NA	
Fixed dystonia of neck	1	NA	
Functional palatal tremor	1	NA	
Functional weakness	3	NA	

### Procedure and experimental design

Participants were seated in a comfortable armchair with hands relaxed on the armrest of the chair and their eyes closed. SEPs were elicited by electrical stimulation of the right median nerve at the wrist using a constant current square-wave pulse (0.2 ms duration). The anode was placed over the median nerve at the wrist and the cathode 2 cm proximal to the anode. The intensity used was the motor threshold for each subject. EEGs were recorded over the scalp on the left with three Ag/AgCl scalp electrodes at three sites on according to the International 10–20 System (F3, C3, and P3). The reference electrode was placed on the right mastoid and the ground on the left mastoid. Electrode impedance was monitored regularly during the course of the experiment and was kept less than 5kΩ. Surface EMG of right abductor pollicis brevis (APB) was monitored simultaneously.

SEPs were recorded in two conditions presented in a randomised order in a single session. In the rest condition, the subjects were relaxed and instructed not to react to the stimulus which was delivered at a frequency of 2.1Hz. In the movement condition, they were instructed to make a self-paced abduction movement of the right thumb. When the EMG signal recorded from the APB rose above 0.15 microvolts the median nerve stimulus was triggered, thus recording an SEPs at the onset of movement. For each condition, 500 traces were recorded. Each trace lasted 470ms. During recording, the sampling rate was set at 2000Hz, and data were online filtered with 20-3000Hz band-pass filter (CED1401 plus, Cambridge Electronics design, Cambridge, UK), averaged and stored in a computer for off-line analysis. Trials with artefacts exceeding 100μV were manually rejected. The order of the two sessions was counterbalanced across subjects.

### Data analysis

We made measurements on the following SEPs components: N30 at frontal electrode and N20 at central and parietal electrodes. For the identification of the amplitudes, we used the following criteria. N30 was defined as the peak-to-peak values at frontal lead formed between 23–33 ms after the sensory stimulus was given. We measured N20 as the peak-to-peak values between 14 and 22ms after the onset of the sensory stimulation at central and parietal electrodes.

SPSS Statistics software (version 21.0.0) was used for the statistical analysis. Our measure of SEPs suppression was the ratio between SEPs amplitude at the onset of movement and baseline SEPs amplitude, and was analysed for the three EEG components. Normality of errors was assessed using the Kolmogorov-Smirnoff test. When not normally distributed, the data were subjected to a Log10 transformation. P-values for categorical variables were calculated with the use of Fisher's exact test. We conducted a repeated measures multi-way analysis of variance (ANOVA) with the following factors: SEPs COMPONENTS (F3, C3, P3) and GROUP (patients vs. healthy controls).Post-hoc tests were conducted with Bonferroni corrections for multiple comparisons. P values less than 0.05 were considered to be significant.

## Results

Demographic characteristics of the participants are shown in Table 1. Clinical features included an acute onset and rapid progression of the symptoms after a minor peripheral injury. Most had an immediate dramatically positive response to Botulinum Toxin injections and, typically, they had a history of sudden and spontaneous remissions and relapses.

The amplitudes and latencies of evoked potentials in control and patient groups in the rest condition were not different (Table 2).

**Table 2:** Amplitudes and latencies of representative SEPs peaks at rest in patients and controls.

		controls	patients	t -value	p-value
**Amplitude (μV)**	**N30 (F3)**	2.31±0.96 [0.23]	1.97±1.23 [0.30]	0.91	>0.05
	**N20 (C3)**	2.77±1.17 [0.28]	2.43±1.14 [0.28]	0.83	>0.05
	**N20 (P3)**	2.76±1.24 [0.30]	2.48±1.22 [0.30]	0.67	>0.05
**Latency (ms)**	**N30 (F3)**	28.64±1.73 [0.41]	28.76±0.83 [0.20]	-0.25	>0.05
	**N20 (C3)**	17.42±0.94 [0.22]	18.01±1.27 [0.31]	-1.53	>0.05
	**N20 (P3)**	17.88±0.93 [0.22]	18.17±1.01 [0.24]	- 0.88	>0.05

Values are mean ± standard deviation [standard error of the mean].

**Fig 1:**
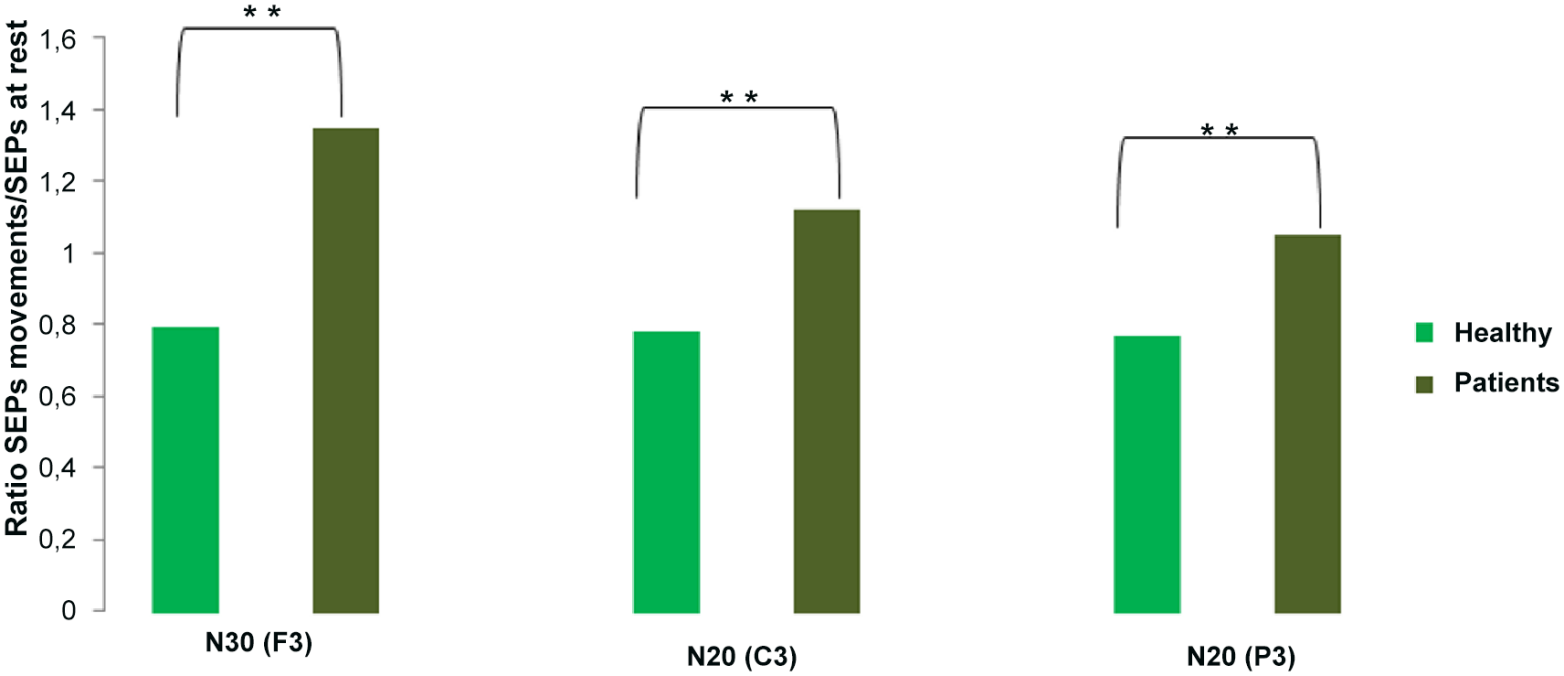
Ratio of SEPs amplitude at the onset of movement and at rest in patients and controls.

We first analysed our measure of SA (ratio between SEPs amplitude at onset of movement and at rest) in healthy controls and patients with FMD and explored whether there were differences. A repeated measures ANOVA with SEPs COMPONENTS (F3, C3 and P3) as within-subjects factors and GROUP as between-subjects factor revealed that there was a significant main effect of GROUP (F(1,21) = 0.88, p = < 0.001). Post Hoc exploration of this effect revealed that this was due to an absence of SEPs suppression in patients (ratio > 1for all the SEPs components) compared to healthy controls, who had SEPs suppression (ratio < 1 for all the SEPs components) (“Fig 1”). A significant difference was found between the two groups for the ratio SEPs movement onset/rest in each electrode: 0.79 in healthy controls and 1.35 in patients at F3 (p < 0.001, t = -4.22), 0.78 in healthy controls and 1.12 at patients C3 (p = 0.004, t = -3.15) and 0.77 in healthy controls and 1.05 at patients P3 (t = -2.88, p = 0.007). We found no significant effect of SEPs ELECTRODE (p = 0.34) nor a SEPs ELECTRODE x GROUP (p = 0.11) interaction.

## Discussion

In this study we have explored the physiological phenomenon of SEPs suppression at the onset of movement in patients with FMD. We have found that patients with FMD did not display attenuation of N20 and N30 amplitudes at the onset of movement compared to healthy controls. Suppression of SEPs around the onset of a voluntary movement is a well described physiological phenomenon [11], and is plausibly the electrophysiological correlate of the psychophysiological reduction in intensity of self-generated sensory stimuli probed by previously described tasks such as the force matching task [9]. Indeed, the attenuation of the intensity of sensations and their electrophysiological/functional imaging correlates when they are self-generated is a widely reported phenomenon across sensory domains [12,13]. The link between sense of agency and SA has been made in various studies in healthy people and in clinical populations including patients with schizophrenia and people with delusional beliefs [14]. The proposal is that SA reflects a “labelling” of sensations (including those produced by movement) as self-generated, and if this is reduced, then it is more likely that sensations will be inferred to be externally generated and lack a sense of agency for their production.

Movement abnormalities in patients with FMD have features that one would typically associate with a strong sense of “voluntariness”, in particular the requirement for attention for the abnormal movement to occur and the resolution of the abnormal movement with distraction of attention. However, patients report the movements to be out of their control. This paradox likely underlies much of the ambivalence about the “genuineness” of FMD. We have previously used a force-matching task to compare SA in patients with FMD and healthy controls, and found a reduction in SA in patients with FMD. Here we extend these findings by exploring an electrophysiological correlate of movement which is plausibly linked to SA as measured by the force-matching task. We therefore have converging evidence for a dysfunction in a specific aspect of the physiology of motor control, and intriguingly one which has been linked to the process of conferring sense of agency for movement.

In contrast to the force matching task, the paradigm used in the present study is technically easy with only simple equipment required and a short experimental time. There is a need for biomarkers of functional motor symptoms, for example for use in clinical trials of (novel) treatments. If these results can be confirmed, and importantly if normalisation of this measure of SA can be shown to occur with improvement of symptoms with treatment, then this could be a suitable biomarker for future studies. We do not suggest that sensory attenuation (at least as we have tested it here) is likely to be a useful diagnostic biomarker of FMD. We think it is likely that there will be abnormalities of sensory attenuation in “organic” movement disorders, though the mechanism of this abnormality will, we suggest, be fundamentally different from the one we propose for FMD.

We acknowledge some limitations to our study. First, the sample size is small and we cannot exclude that in a larger cohort data may have greater statistical efficiency. However, we chose patients with clinically typical FMD using standardized criteria and believe that they do accurately represent patients with this diagnosis. Furthermore, the fact that we were able to show a significant group effect with a relatively small sample size suggests the effect sizes in question are relatively large. Second, as in our previous study on force matching [9],we did not include patients with functional tremor, the most common FMD, because tremor commonly involves upper limbs and this was an exclusion criterion for the study. Third, we have speculated that suppression of SEPs at the onset movement is the electrophysiological correlate of the phenomenon of SA assessed by a force-matching paradigm, but we did not directly compare the two phenomena in this study, and therefore this remains a speculative interpretation. Fourth, as in our previous force matching study, we deliberately assessed clinically unaffected body parts. We therefore cannot demonstrate via this study whether the phenomenon we observed is a trait of patients with FMD (or related to other co-morbidities) or is genuinely related to the state of having a functional movement disorder.

In conclusion, these results confirm a deficit in SA for self-paced movement in patients with FMD, which could be plausibly linked to a disruption in sense of agency for movement. The measurement of SA in this relatively simple paradigm is an interesting candidate biomarker for FMD which could be explored in future work.
